# 749 Clinical Decision Support Systems, Electronic Health Records and Fluid Resuscitation: Harnessing the Power of Technology

**DOI:** 10.1093/jbcr/irae036.291

**Published:** 2024-04-17

**Authors:** Bowen Pan, Stacey Richerbach, Floredes Menodiado, Robert McGuire, Tiffany Hockenberry, Karen J Richey, Kevin N Foster

**Affiliations:** Arizona Burn Center, Chandler, AZ; Arizona Burn Center Valleywise Health, Phoenix, AZ; Epic, Verona, WI; Valleywise Health, Phoenix, AZ; Arizona Burn Center, Chandler, AZ; Arizona Burn Center Valleywise Health, Phoenix, AZ; Epic, Verona, WI; Valleywise Health, Phoenix, AZ; Arizona Burn Center, Chandler, AZ; Arizona Burn Center Valleywise Health, Phoenix, AZ; Epic, Verona, WI; Valleywise Health, Phoenix, AZ; Arizona Burn Center, Chandler, AZ; Arizona Burn Center Valleywise Health, Phoenix, AZ; Epic, Verona, WI; Valleywise Health, Phoenix, AZ; Arizona Burn Center, Chandler, AZ; Arizona Burn Center Valleywise Health, Phoenix, AZ; Epic, Verona, WI; Valleywise Health, Phoenix, AZ; Arizona Burn Center, Chandler, AZ; Arizona Burn Center Valleywise Health, Phoenix, AZ; Epic, Verona, WI; Valleywise Health, Phoenix, AZ; Arizona Burn Center, Chandler, AZ; Arizona Burn Center Valleywise Health, Phoenix, AZ; Epic, Verona, WI; Valleywise Health, Phoenix, AZ

## Abstract

**Introduction:**

Electronic Health Record (EHR) raw data, in conjunction with analysis via Clinical Decision Support Systems (CDSS) has great potential to enhance bedside decision-making and improve patient outcomes. However, technology systems are often siloed, limiting interoperability, and creating inefficient workflows. Proprietary software is costly, lacks transparency and limits customization. The purpose of this project was to incorporate a custom burn fluid resuscitation CDSS into our existing EHR and evaluate safety and efficacy.

**Methods:**

Following Institutional Review Board approval, a team was formed of representatives from the EHR vendor, local health informaticists, burn surgeons, burn nurses, and research staff. An initial equation using the Parkland formula as a starting point was created and run in the background during resuscitation of patients over the course of several months. The formula went through a feedback-based refinement cycle to better align with historical datasets and expert opinion. Following implementation, safety data was analyzed after each patient and then cumulatively compared to historical controls.

**Results:**

A total of 17 adults were resuscitated using the CDSS, excluded from analysis were patients that had comfort care initiated within the first 12 hours. There were no significant differences for age, weight or TBSA between CDSS (n=13) and controls (CON) (n=138). The average ml/kg/TBSA in CDSS was 5.43 vs 6.49 CON (p=.259) and duration of resuscitation was 26.31 hours CDSS vs 31.83 hours CON (p< 0.0867). Mortality between groups was not significantly different CDSS 23% vs CON 24% (p=0.125). Several process improvements were realized, including availability of CDSS data at all workstations, full integration of data within the EHR thus providing one “source of truth,” the capability to have uninterrupted resuscitation across domains of care, enhanced efficiency in documentation by nursing, and the ability to have ongoing monitoring of safety and efficacy.

**Conclusions:**

This proof-of-concept project demonstrates the feasibility of developing tools, namely burn fluid resuscitation, within the EHR that are user-centric and have fully integrated workflows. To date, no safety signals have been detected. Holding creative rights to the algorithm enables us to continue to refine the formula based on evolving evidence. With the future in mind, the CDSS is designed to incorporate data from automated IV infusion and urine output monitoring systems. The project provides a framework for the customization of additional Clinical Decision Support System projects, providing a powerful platform to drive quality improvement initiatives.

**Applicability of Research to Practice:**

The framework for this project has outstanding potential to revolutionize how burn resuscitation care is provided and documented at the bedside.

